# Directing Diarrhoeal Disease Research towards Disease-burden Reduction

**DOI:** 10.3329/jhpn.v27i3.3374

**Published:** 2009-06

**Authors:** Margaret Kosek, Claudio F. Lanata, Robert E. Black, Damian G. Walker, John D. Snyder, Mohammed Abdus Salam, Dilip Mahalanabis, Olivier Fontaine, Zulfiqar A. Bhutta, Shinjini Bhatnagar, Igor Rudan

**Affiliations:** ^1^ Department of International Health, Johns Hopkins Bloomberg School of Public Health, Baltimore MD, USA; ^2^ Instituto de Investigación Nutricional, Lima, Peru; ^3^ Medical School, Peruvian University of Applied Sciences, Lima, Peru; ^4^ Department of Pediatrics, UCSF School of Medicine, San Francisco, CA, USA; ^5^ ICDDR,B, GPO Box 128, Dhaka 1000, Bangladesh; ^6^ Society for Applied Studies, CF-198, Salt Lake, Sector 1, Kolkata 700 064, West Bengal, India; ^7^ Division of Child and Adolescent Health, World Health Organization, Geneva, Switzerland; ^8^ Aga Khan University, Karachi, Pakistan; ^9^ All India Institute of Medical Sciences, New Delhi, India; ^10^ Croatian Centre for Global Health, University of Split Medical School, Croatia; ^11^ Department of Public Health Sciences, University of Edinburgh Medical School, Scotland, UK

**Keywords:** Child heath, Diarrhoeal diseases, Mortality, Priority setting, Medical research

## Abstract

Despite gains in controlling mortality relating to diarrhoeal disease, the burden of disease remains unacceptably high. To refocus health research to target disease-burden reduction as the goal of research in child health, the Child Health and Nutrition Research Initiative developed a systematic strategy to rank health research options. This priority-setting exercise included listing of 46 competitive research options in diarrhoeal disease and their critical and quantitative appraisal by 10 experts based on five criteria for research that reflect the ability of the research to be translated into interventions and achieved disease-burden reduction. These criteria included the answerability of the research questions; the efficacy and effectiveness of the intervention resulting from the research; the maximal potential for disease-burden reduction of the interventions derived from the research; the affordability, deliverability, and sustainability of the intervention supported by the research; and the overall effect of the research-derived intervention on equity. Experts scored each research option independently to delineate the best investments for diarrhoeal disease control in the developing world to reduce the burden of disease by 2015. Priority scores obtained for health policy and systems research obtained eight of the top 10 rankings in overall scores, indicating that current investments in health research are significantly different from those estimated to be the most effective in reducing the global burden of diarrhoeal disease by 2015.

## INTRODUCTION

Diarrhoeal disease causes an estimated 1.8 million deaths per year (1). Despite evidence of reduction in mortality over the last 50 years (2,3), diarrhoeal disease continues to be a major killer of children aged less than five years and a principal cause of morbidity for most impoverished children of the world. It is well-known that most of these deaths are preventable with existing disease- control strategies (4).

Although there are numerous causes for the lack of greater progress in the control of diarrhoeal diseases, it is clear that our investments in related research over the last 20 years have not had the greatest attainable impact. It is now increasingly recognized that research priorities do not optimally address the needs of children in developing countries (5,6). Setting research priorities is clearly a challenging and imperfect process that relies on the best data available and the knowledge of experts in the field to fill in knowledge gaps. Clearly, data regarding the number and cause of deaths and the coverage of interventions are limited and imperfect. How to integrate available data with expert opinion is an evolving area. Classically, they were derived from expert group meetings and, more recently, Delphi exercises have been employed as an improved strategy to incorporate expert opinion in decision-making processes. Cost-effectiveness analyses have been used for prioritizing among health interventions but have not been systematically used for determining the most promising research. In any case, cost-effectiveness analysis would have limited usefulness without a critical assessment of the likelihood that investments in research would result in reduction in the burden of disease. The Child Health and Nutrition Research Initiative (CHNRI) was founded to encourage and support research on the important child health problems in low- and middle-income countries. The CHNRI developed a structured process that was designed to measure the likelihood that funding-specific research questions would be successful in reducing child morbidity and mortality. This novel methodology was used here for assessing the priority for funding particular avenues of research to address the burden of disease caused by childhood diarrhoea (7,8).

## MATERIALS AND METHODS

### Selection of group members and research options

As part of a larger exercise of assessing research priorities for major child health conditions, the CHNRI decided that the exercise should consider research for burden of reduction of diarrhoeal disease by 2015 among children aged less than five years. Since the currently-accepted disease-burden measure is the disability-adjusted life-year (DALY), which incorporated both mortality and morbidity components of disease, our scoring exercise included both mortality and morbidity components. However, due to the current thinking on disease weighting, most burden of diarrhoeal disease is related to mortality rather than morbidity, and scorers were asked to respect this current thinking. The CHNRI Secretariat selected two group members (CL and MK). These two members defined the list of research options in communication with IR, based on a systematic framework for listing research options relating to a single disease developed by the CHNRI (7,8). This systematic approach enables comprehensive listing and equal treatment of research options in different broad research domains: epidemiologic research, health policy and systems research (HSPR), research intended to improve existing interventions, and research to develop new interventions. The list of research questions was intentionally limited to less than 50 to allow individuals to be able to complete the scoring process in a single day. These research options included different strategies in diarrhoeal disease control encompassing those aimed at improving water and sanitation infrastructure, those targeting healthcare-delivery strategies, those addressing nutritional deficiencies, and research to evaluate novel diagnostics and vaccines. The research options were then categorized as either: (a) Health policy and systems research (HSPR) that aimed to improve the efficiency and coverage of known interventions; (b) Research that improved existing interventions by making them more affordable or deliverable; or (c) Research options to develop entirely new interventions. Although a research option could encompass multiple different research questions, it was made sufficiently narrow in scope to be able to anticipate specific research project derivatives that could be evaluated by the scoring process.

A further 17 experts were invited to participate, of whom eight completed and returned priority scores for a total group of scorers comprising 10 individuals [Group of scorers: Shinjini Bhatnager, Zulfiqar A. Bhutta, Olivier Fontaine, Margaret Kosek, Claudio F. Lanata, Dilip Mahalanabis, Mohammed Abdus Salam, John D. Snyder, Cesar Victora, and Damian G. Walker].

These individuals were categorized as physicians with expertise in infectious diseases, gastroenterologists, public-health researchers specialized in programmatic issues, a health economist, and public-health researchers in areas other than programme development and evaluation. Each one scored the individual research options independently using a five-component structured model developed by the CHNRI to evaluate health research. The five components consist of the following: (a) likelihood that the research option can yield new knowledge in an ethical manner; (b) likelihood that the research findings will lead to efficacious and effective interventions; (c) likelihood that the intervention derived from the research would be affordable and deliverable to the population of interest; (d) most likely maximum burden of disease reduction that could be derived from interventions resulting from research within the option; and (e) likely impact that the derivates of the research will have on equity.

Scores were computed as percentage of maximal obtainable points for each of the major five components being evaluated and then combined for an overall score. The scores outline both limitations and strengths of each research option. Each of the five intermediate scores reflect the likelihood that the research option will be answerable, that it will result in an effective intervention, that the resulting intervention will be deliverable, that the resulting intervention will increase equity, and an estimation of the maximal disease-burden impact an intervention resulting from the research is foreseen to have. When added together, this overall score becomes a quantitative measure of the collective optimism that research in that area can have substantial impact prior to 2015. Although this system easily accommodates weighting of these five options by donors or regional agencies or other stakeholders, we have presented the unweighted results. The process of scoring is presented in greater detail elsewhere (7-10).

## RESULTS

The listing of research options yielded 46 options. Twenty-one research options were designed as health policy and systems research to increase the efficiency of interventions already in place, 10 options addressed research to improve the affordability and deliverability of known interventions, and 15 options were primary research to develop new interventions. The complete list of the 46 research options is presented in Table 1. The questions guiding the scoring of the research options by each criteria are shown in Table 2. An excel file that facilitates the scoring exercise by providing a spreadsheet for the input of scores is available online (http://www.icddrb.org/jhpn).

**Table 1.** List of 46 research options scored by diarrhoeal disease experts

Research option	
RO1:	Health policy and systems research (HPSR) to increase access to ORS packets at all times in all sites for all children who may need it
RO2:	Research to generate new knowledge (mostly effectiveness studies) to increase the use of low-osmolarity ORS
RO3:	Health policy, systems, and education/behaviour modification research to increase the percentage of infants with exclusive breastfeeding at <6 month of age
RO4:	Health policy, systems, and education/behaviour modification research to increase the percentage of infants and children, aged less than 2 years, who are breastfed
RO5:	Health system research to increase the coverage of measles vaccine
RO6:	HPSR to improve the coverage of rotavirus vaccine in countries with the greatest needs
RO7:	Systems and education/behaviour modification research to increase water consumed per person per day
RO8:	System research to measure the effectiveness of piped water systems on diarrhoea if they are installed at the community vs in the home
RO9:	System research to measure the effectiveness of piped water systems on diarrhoea if they are installed so as to provide intermittent vs 24-hour availability
RO10:	Systems and education/behaviour modification research to increase the coverage of sewage systems
RO11:	Systems and education/behaviour modification research to increase the prevalence of effective latrines
RO12:	Health policy, systems, and education/behaviour modification research to increase the proportion of women and children washing their hands effectively to improve hand-washing promotion
RO13:	Education/behaviour modification research to increase the energy density of weaning foods at the household level (in areas with food availability)
RO14:	HPSR to allow that all mothers with a child with diarrhoea will know how to recognize danger-signs for timely referral/self-referral of severe cases
RO15:	HPSR to improve the quality of care of moderate/severe diarrhoea cases through standardized case management
RO16:	HPSR to improve prescription of appropriate antibiotics for dysentery
RO17:	Efficacy/effectiveness studies of interventions of behaviour modification to reduce baby bottle-use
RO18:	Education/behaviour modification research to increase the use of refrigerators for storage of weaning foods
RO19:	Efficacy/effectiveness studies and education/behaviour modification research to increase consumption of *Lactobacillus* GG probiotic
RO20:	Efficacy/effectiveness studies of interventions of behaviour modification to increase potties-use/improved faece-disposal practices
RO21:	HPSR to generate new knowledge to increase the coverage of vitamin A supplementation
RO22:	System and community research to reduce costs/improve deliverability and increase the coverage of piped water systems
RO23:	Effectiveness, costs, sustainability, system and behavioural modification/cultural research to increase the use of point-of-use water disinfection: implementation of point-of-use treatment and water-storage practices
RO24:	Research to develop new ways of sewage-treatment systems that will make them affordable to developing countries
RO25:	Research to improve the deliverability, measure effectiveness, and determine the sustainability of fly-control interventions
RO26:	Effectiveness studies and studies that will reduce the cost/improve the deliverability of cholera vaccines in high-burden countries
RO27:	Cash-transfer programmes to improve diet quality and nutrition in poor areas
RO28:	Policy, systems, and education/behaviour modification research to improve current strategies aiming at improving the quality of diet of family in areas with low access to good diets
RO29:	Effectiveness, HPSR, and educational/behaviour modification studies to improve the deliverability/cost of zinc treatment in diarrhoea-control programmes in several regions of the world with different epidemiological profiles
RO30:	Efficacy, effectiveness and cost studies that will increase the use of zinc food-fortification programmes in developing countries
RO31:	Cost-effectiveness studies of rotavirus vaccine in different epidemiologic contexts
RO32:	Develop norovirus vaccines
RO33:	Develop *Shigella* vaccines
RO34:	Develop ETEC vaccines
RO35:	Develop *Campylobacter* vaccines
RO36:	Develop EPEC vaccines
RO37:	Develop *Helicobacter pylori* vaccines
RO38:	Develop vaccines for *Entamoeba histolytica*
RO39:	Develop new measles vaccines that will be heat-stable and able to immunize newborns
RO40:	Solar ovens to keep weaning foods above >50 °C for a day
RO41:	Low cost, no electrical/no fuel consuming refrigerators to storage food at the household level
RO42:	New antibiotics for drug-resistant *Shigella*
RO43:	New antibiotics for drug-resistant cholera
RO44:	Develop interventions that will reduce bacterial contamination of crops irrigated with contaminated water in developing countries
RO45:	Further development of antisecretory agents in the management of paediatric diarrhoea
RO46:	Develop the technology to deliver zinc to children using prolong dosing intervals

EPEC=Enteropathogenic *Escherichia coli* ETEC=Enterotoxigenic *Escherichia coli* ORS=Oral rehydration solution

RO=Research option

**Table 2.** Questions with which to assess the 5 criteria for each selected research option. For an interactive spreadsheet for scoring, see website (http://www.icddrb.org/jhpn)

Scoring criteria	Question 1	Question 2	Question 3
Criteria 1: Likelihood that the research will lead to new knowledge in an ethical way	1.1 Would you say the research question is well-framed and endpoints are well-defined?	1.2 Based on: (a) the level of existing research capacity in proposed research and (b) the size of the gap from current level of knowledge to proposed endpoints, would you say that a study can be designed to answer the research question and to reach the proposed endpoints of the research?	1.3 Do you think that a study needed to answer the proposed research question would obtain ethical approval without major concerns?
Criteria 2: Assessment of the likelihood that the intervention which would be developed through the research would be efficacious	2.1 Based on the best existing evidence and knowledge, would the intervention which would be developed/improved through proposed research be efficacious?	2.2 Based on the best existing evidence and knowledge, would the intervention which would be developed/improved through the proposed research be effective?	2.3 Would you say that the evidence upon which these opinions are based (answers to prior 2 questions) is of high quality?
Criteria 3: Likelihood that the intervention based on the research would be affordable, deliverable, and sustainable in the population of interest	3.1 Taking into account the level of difficulty with intervention delivery from the perspective of the intervention itself (e.g. design, standardizability, safety), the infrastructure required (e.g. human resources, health facilities, communication and transport infrastructure) and users of the intervention (e.g. need for change of attitudes or beliefs, supervision, existing demand), would you say the endpoints of the research would be deliverable within the context of interest?	3.2 Taking into account the resources available to implement the intervention, would you say that the endpoints of the research would be affordable within the context of interest?	3.3 Taking into account government capacity and partnership requirements (e.g. adequacy of government regulation, monitoring, and enforcement; governmental intersectoral coordination, partnership with civil society and external donor agencies; favourable political climate to achieve high coverage), would you say that the endpoints of the research would be sustainable within the context of interest?
Criteria 4: Assesment of the maximal potential for disease-burden reduction	4.1 Taking into account the results of conducted intervention trials, or for the new interventions the proportion of avertable burden under an ideal scenario, would you say that the sucessful reaching of research endpoints would have the capacity to remove 5% of the burden or more?	4.2 To remove 10% or more?	4.3 To remove 15% or more?
Criteria 5: Assessment of the impact of proposed research on equity	5.1 Would you say that the present distribution of the disease burden affects mainly the underpriveleged in the population?	5.2 Would you say that either (a) mainly the underpriveleged, or (b) all segments of the society equally would be the most likely to benefit from the results of the proposed research after its implementation?	5.3 Would you say that the proposed research has the overall potential to improve equity in disease-burden distribution in the long term (e.g. 10 years)?

### Criterion 1: Generating new knowledge

Priority scores for research options evaluated solely on the criterion of their ability to generate new knowledge ranged from 57.0% to 95.8%. The top five research options that were predicted to encounter minimal obstacles in their realization are listed in Table 3. The research option that received the highest score for this criterion was the conduction of cost-effectiveness studies of rotavirus vaccines in different epidemiologic contexts. The second ranked research option by this criterion was effectiveness studies to evaluate the expanded use of low-osmolarity oral rehydration solution (ORS). Two research alternatives received equal scores to rank third. These options included health policy and systems research to improve the deliverability and cost of zinc treatment in diarrhoeal disease programmes and health policy and systems research to improve coverage of rotavirus vaccine in countries with the greatest burden of disease. Health policy and systems research to improve case management of moderate and severe cases of diarrhoeal illness by using standardized case management and the development of new antibiotics for the treatment of drug-resistant shigellosis tied for fourth place in the ranking. The fifth rank was occupied by two evenly-ranked options of health policy and services research to increase access to ORS envelops at all times to all children who need it and health policy and services research to increase the percentage of infants exclusively breastfed up to the age of six months.

**Table 3.** Top 5 options for generating new knowledge (Criterion 1)

Rank	Category	Research option	Score (%)
1	3	RO31: Cost-effectiveness studies of rotavirus vaccine in different epidemiologic contexts	95.8
2	2	RO2: Research to generate new knowledge (mostly effectiveness studies) to increase the use of low-osmolarity ORS	88.3
3	3	RO29: Effectiveness, health policy, and systems research (HPSR) and educational/behaviour modification studies to improve the deliverability/cost of zinc treatment in diarrhoea-control programmes in several regions of the world with different epidemiological profiles	86.7
3	2	RO6: HPSR to improve coverage of rotavirus vaccine in countries with the greatest needs (impact on morbidity and mortality)	86.7
4	2	RO15: HPSR to improve the quality of care for moderate and severe diarrhoea cases through standardized case management (impact on mortality only)	85.2
4	4	RO42: New antibiotics for drug-resistant enteropathogens: *Shigella*	85.2
5	2	RO1: HPSR to increase access to ORS packets at all times in all sites for all children who may need it	85.0
5	2	RO3: Health policy, systems, and education/behaviour modification research to increase percentage of infants with exclusive breastfeeding <6 months of age	85.0

ORS=Oral rehydration solution

RO=Research option

Low scoring research options in this category were predominated by enteric vaccines that are currently in early stages of development as these are unlikely to yield clinical trials demonstrating efficacy prior to 2015. Scores for *Campylobacter*, enteropathogenic *Escherichia coli, Entamoeba histolytica,* and norovirus all obtained scores for answerability ranging between 57% and 68%.

### Criterion 2: Efficacy and effectiveness

The priority scoring of research options based solely on their predicted potential to lead to (or improve) efficacious and effective interventions yielded scores ranging from 32.0% to 97.9% (Table 4). The top research option when judged by this criterion was the study of cost-effectiveness of rotavirus vaccine in different epidemiologic contexts. The second-ranking research option was health policy and services research to increase the access to ORS for all children who may need it. Effectiveness, health policy and services research, and educational/behavioural modification studies to improve the deliverability and cost of zinc treatment in diarrhoea-control programmes in several regions of the world with different epidemiologic profiles occupied the third rank. Tied for the fourth ranking was research to generate new knowledge (mostly effectiveness studies) to increase use of low-osmolarity ORS and health systems research to increase the coverage of measles vaccine. The fifth-ranking research option in terms of efficacy and effectiveness was health policy and systems research to improve the quality of care of moderate/severe diarrhoea cases through standardized case management.

**Table 4.** Top 5 options for efficacy/efficaciousness (Criterion 2)

Rank	Category	Research options	Score (%)
1	3	RO31: Cost-effectiveness studies of rotavirus vaccine in different epidemiologic contexts	97.9
2	2	RO1: HPSR to increase access to ORS packets at all times in all sites for all children who may need it	93.3
3	3	RO29: Effectiveness, HPSR, and educational/behaviour modification studies to improve the deliverability/cost of zinc treatment in diarrhoea-control programmes in several regions of the world with different epidemiological profiles	90.7
4	2	RO2: Research to generate new knowledge (mostly effectiveness studies) to increase use of low-osmolarity ORS	86.7
4	2	RO5: Health-systems research to increase the coverage of measles vaccine	86.7
5	2	RO15: HPSR to improve the quality of care for moderate and severe diarrhoea cases through standardized case management	85.4

HPSR=Health policy and systems research; ORS=Oral rehydration solution; RO=Research option

Research options that received low scores in this area were diverse and included research relating to fly control, the improved storage of weaning foods, and the development of interventions meant to curb bacterial contamination of crops.

### Criterion 3: Sustainability and deliverability

Priority scores for options evaluated based on sustainability and deliverability alone had the widest variation in obtained scores of the five criteria with scores ranging from 9% to 90%. The highest score in terms of sustainability and deliverability was given to effectiveness studies to increase the use of low-osmolarity ORS which obtained a priority score of 90% (Table 5). The second ranking was to studies relating to the uptake and evaluation of zinc in diarrhea-control programmes in the different epidemiologic contexts. The third ranking was shared by research options for health policy and systems research to improve the prescription of appropriate antibiotics for dysentery and to increase the coverage of vitamin A supplementation. The evaluation of the cost-effectiveness of rotavirus vaccine in the different epidemiologic contexts and health policy and systems research to improve the quality of care of moderate and severe diarrhoea cases through standardized case management were ranked fourth. The fifth-ranking option was health policy, systems and education and behavioural modification research to increase the proportion of women and children washing their hands effectively.

**Table 5.** Top 5 options for sustainability and deliverability (Criterion 3)

Rank	Category	Research option	Score (%)
1	2	RO2: Research to generate new knowledge (mostly effectiveness studies) to increase the use of low-osmolarity ORS	90.0
2	3	RO29: Effectiveness, HPSR, and educational/behaviour modification studies to improve the deliverability/cost of zinc treatment in diarrhoea-control programmes in several regions of the world with different epidemiological profiles	88.9
3	2	RO16: HPSR to improve prescription of appropriate antibiotics for dysentery	85.2
3	2	RO21: HPSR to generate new knowledge to increase the coverage of vitamin A supplementation (to reduce severity of diarrhoea and improve mortality)	85.2
4	3	RO31: Cost-effectiveness of rotavirus vaccine in different epidemiologic contexts	83.3
4	2	RO15: HPSR to improve the quality of care for moderate and severe diarrhoea cases through standardized case management	83.3
5	2	RO12: Health policy, systems, and education/behaviour modification research to increase the proportion of women and children washing their hands effectively	77.8

HPSR=Health policy and systems research; ORS=Oral rehydration solution; RO=Research option

Research options scoring poorly in deliverability and sustainability included options to increase the use of refrigerators for the storage of weaning foods, cash-transfer programmes to improve the quality of diet and nutrition in poor areas, and vaccines for *E. histolytica* and *Camplylobacter*.

### Criterion 4: Maximal potential for diseaseburden reduction

Priority scores to judge the maximum potential for disease-burden reduction ranged from 8% to 79% (Table 6). The top two scoring research options related to use of rotavirus vaccine. The top scoring research option achieved a priority score of 79.2% and was aimed at evaluating the cost-effectiveness of rotavirus vaccination in the different epidemiologic contexts while health policy and systems research to improve the coverage of rotavirus vaccination in countries with the greatest need had a 76.7% priority score. Health policy and services research to increase access to ORS envelops received the third-ranking position and was followed in the ranking by health policy and services research to improve the deliverability and cost of zinc treatment in diarrhoea-control programmes in several regions of the world with different epidemiologic profiles. The fifth-ranking option was health policy and systems research to train mothers in the recognition of danger signs for the timely self-referral of severe cases of diarrhoea.

**Table 6.** Top 5 option for maximal potential to decrease disease burden (Criterion 4)

Rank	Category	Research option	Score (%)
1	3	RO31: Cost-effectiveness of rotavirus vaccine in different epidemiologic contexts	79.2
2	2	R06 : HPSR to improve the coverage of rotavirus vaccine in countries with the greatest needs	76.7
3	2	RO1: Health policy and systems research (HPSR) to increase access to ORS packets at all times in all sites for all children who may need it	75.0
4	3	RO29: Effectiveness, HPSR, and educational/behaviour modification studies to improve the deliverability/cost of zinc treatment in diarrhoea-control programmes in several regions of the world with different epidemiological profiles	70.0
5	2	RO14: HPSR to allow that all mothers with a child with diarrhoea will know how to recognize danger-signs for timely referral/self-referral of severe cases	64.8

ORS=Oral rehydration solution; RO=Research option

Low scores in maximal potential for disease-burden reduction were obtained by research options relating to fly-control interventions, effectiveness, and deliverability of cholera vaccine, and efficacy and effectiveness studies relating to use of probiotic *Lactobacillus* GG.

### Criterion 5: Equity

The scores for equity were the highest of any of the criteria and ranged from 43.0% to 98.3%. All five top options received priority scores greater than 90% (Table 7). The top score was given to health systems research to increase the coverage of measles vaccine. The second-ranking research option was policy, systems, and education and behaviour modification research to improve current strategies aiming at improving the quality of diet of families in areas with low access to good diets. Systems and education and behavioural modification research to increase the prevalence of latrines was ranked third. Educational and behavioural modification research to increase the energy density of weaning foods at the household level in areas with food availability and systems and behavioural research to improve water consumed per person per day obtained equal scores to rank fourth among the selected research options. The fifth-ranking option was health policy and systems research to improve the quality of care of moderate and severe cases of diarrhoea through standardized case management.

**Table 7.** Top 5 options for equity (Criterion 5)

Rank	Category	Research option	Score (%)
1	2	R05 : Health systems research to increase the coverage of measles vaccine	98.3
2	3	RO28: Policy, systems, and education/behaviour modification research to improve current strategies aiming at improving the quality of diet of family in areas with low access to good diets	96.7
3	2	RO11: Systems and education/behaviour modification research to increase the prevalence of effective latrines	93.3
4	2	RO13: Education/behaviour modification research to increase the energy density of weaning foods at the household level (in areas with food availability)	92.6
4	2	RO7: Systems and education/behaviour modification research to improve water consumed per person per day	92.6
5	2	RO15: HPSR to the improve the quality of care for moderate and severe diarrhoea cases through standardized case management	90.7

HPSR=Health policy and systems research; RO=Research option

Low priority scores were obtained by research options to evaluate probiotic *Lactobacillus* GG, new antisecretory agents, and vaccines for *E. histolytica*, norovirus, and *H. pylori*.

#### Combined results

The overall priority scores that were assigned to research options by computing unweighted means of the five intermediate scores ranged from 35.0% to 85.2% (Table 8). The top scoring research items, overall, were predominantly (7/10) options that aimed at increasing the efficiency and coverage of interventions with known effectiveness. The research option receiving the highest priority score addressed effectiveness, health policy and services research to improve the deliverability and cost of zinc treatment in diarrhoea-control programmes in areas with different epidemiologic profiles. The second-ranking research option, which proposed conducting cost-effectiveness studies of rotavirus vaccine in different epidemiologic contexts received a priority score less than 1% lower than the top-ranking option. Health policy and services research to increase access to ORS envelops and to improve the quality of care of moderate to severe cases of diarrhoea through standardized case management were ranked third and fourth respectively. Effectiveness studies to increase the use of low-osmolarity ORS received the fifth-ranking priority score and was followed closely by health policy, systems, and education and behavioural modification research to increase the percentage of infants exclusively breastfed for the first six months of life. Health systems research to increase the coverage of measles and rotavirus vaccines were ranked seventh and eighth respectively. Health policy and systems research to aid mothers in the recognition of danger-signs in cases of severe illness to improve self-referral ranked ninth, and the tenth priority ranking was assigned to efficacy, effectiveness and costing studies to evaluate the increased use of zinc-fortification programmes in developing countries.

**Table 8.** Top 10 research options overall by five criteria

Rank	Category	Research option	Score (%)
1	3	RO29: Effectiveness, HPSR, and educational/behaviour modification studies to improve the deliverability/cost of zinc treatment in diarrhoea-control programmes in several regions of the world with different epidemiological profiles	85.2
2	3	RO31: Cost-effectiveness studies of rotavirus vaccine in different epidemiologic contexts	85.0
3	2	RO1: Health policy and systems research (HPSR) to increase access to ORS packets at all times in all sites for all children who may need it	81.6
4	2	RO15: HPSR to improve the quality of care for moderate/severe diarrhoea cases through standardized case management	80.0
5	2	RO2: Research to generate new knowledge (mostly effectiveness studies) to increase the use of low-osmolarity ORS	78.7
6	2	RO3: Health policy, systems, and education/behaviour modification research to increase the percentage of infants with exclusive breastfeeding <6 months of age	77.4
7	2	RO5: Health systems research to increase the coverage of measles vaccine	77.2
8	2	RO6: HPSR to improve the coverage of rotavirus vaccine in countries with the greatest needs	75.0
9	2	RO14: HPSR to allow that all mothers with a child with diarrhoea will know how to recognize danger-signs for time referral/self-referral of severe cases	74.4
10	3	RO30: Efficacy, effectiveness and cost studies that will increase the use of zinc food-fortification programmes in developing countries	73.0

HPSR=Health policy and systems research; ORS=Oral rehydration solution; RO=Research option

#### Vaccines

It is worth noting that the development of new vaccines for the improved control of the burden of diarrhoeal disease obtained low scores (Table 9). The highest-scoring vaccine was a measles vaccine that would be both heat-stable and effective when administered in the neonatal period. Research directed towards obtaining this goal received a priority score of 65.1%. The highest-scoring vaccine directly targeting an enteric organism was a vaccine against *Shigella*, which obtained a priority score of 63.0%. Determining the cost-effectiveness of the currently-available cholera vaccine received a score of 61.7%.

**Table 9.** Unweighted priority scores (%) for development of novel vaccines to diminish the burden of diarrhoeal disease

Vaccine	Criterion 1(%)	Criterion 2(%)	Criterion 3(%)	Criterion 4(%)	Criterion 5(%)	Overall priority score (%)
New measles vaccines that will be heat-stable and able to immunize						
newborns	76.7	77.8	59.3	50.0	61.7	65.1
*Shigella*	83.3	83.3	44.4	35.2	68.5	63.0
ETEC	66.7	63.0	37.0	42.6	68.5	55.6
EPEC	63.3	43.8	25.0	37.0	68.5	47.5
Norovirus	68.3	54.2	31.3	29.2	44.4	45.5
*Helicobacter pylori*	63.3	41.7	24.1	14.8	42.6	37.3
*Entameoba histolytica*	63.3	43.8	14.6	8.3	55.6	37.1
*Campylobacter*	56.7	41.7	12.5	13.0	51.9	35.1

EPEC=Enteropathogenic *Escherichia coli*; ETEC=Enterotoxigenic *Escherichia coli*

There is little question that the group of scorers was not biased against vaccines as a whole or enteric vaccines. Two of the overall top 10 research options reflected research regarding the appropriate usage of rotavirus vaccine in different settings. In addition to this, health policy and service research to increase the coverage of the current measles vaccine was ranked seventh overall. In general, the existing vaccines with proven efficacy and potential for expanded access were scored higher than theoretical possibilities in vaccine research. Furthermore, as the time set to expect the benefits of the research in terms of disease-burden reduction is 2015, this timetable challenges vaccine research in areas in which knowledge on basic science regarding the basis of acquired immunity or the global burden of disease is lacking. However, even with an efficacy of 95% in preventing mortality and 60% in preventing morbidity, few enteric pathogens cause an aetiologic fraction of morbidity and mortality of diarrhoeal disease large enough to compare with interventions that are independent of aetiology, and scores for criterion 4 (maximum disease-reduction fraction) were, therefore, correspondingly low. While the aetiologic fractions may vary somewhat by region, few pathogens cause more than 10% of incident episodes globally, putting a practical ceiling on the overall diarrhoeal disease-burden reduction available by their control even with high efficacy and coverage. Further stifling the scoring were issues regarding to deliverability, and to a lesser extent, equity.

#### Water and sanitation

It is somewhat surprising to see the predominantly intermediate ranks received by the research options that could broadly be categorized as dealing with water and sanitation, the theoretical mainstay to control the transmission of enteric diseases. Research options covered a broad range of issues, including those that deal with quality and quantity of water (research option 7, 8, 9, 22, and 23; research options addressing the improved disposal of human excreta (research option 10, 11, 24, and 44); and a single research option addressing handwashing (research option 12). As a group, these obtained high scores in terms of equity and answerability and scores predominantly in the second quartile for maximum disease-burden reduction but lower scores in other categories, including deliverability and sustainability.

#### Nutrition

Improving nutritional status has a key role in decreasing the burden of diarrhoeal disease. Two of the top 10 priority scores, overall, went to research options that included zinc, a micronutrient that has shown effects in decreasing the duration of diarrhoeal episodes and the risk of developing persistent diarrhoea. Increasing the coverage of vitamin A supplementation and increasing the energy-density of weaning foods in areas with food availability received priority scores of 71.2% and 67.5% respectively and were in the top quartile of rankings. Research to improve the quality of diet in areas with limited food availability obtained a more modest priority score of 61.0%. Cash-transfer programmes designed to improve diet in areas with restricted access were given a priority score of only 47.1% hampered in large part by the perceived problems in delivery and maintenance of the programmes.

## DISCUSSION

The research options that appear on the list are not comprehensive. These are rather meant to be a selection of the top research options representative of various strategies of diarrhoeal disease control. These include a balance between the development of new interventions with research that addresses improved implementation of interventions that have already been shown to be efficacious. We feel that the structured nature of the process of scoring research options leads to a significant improvement from previously-used methods involving expert consensus. The output is a list of ranking options developed by technical experts scoring independently to avoid problems associated with group dynamics in decision-making. The structure of the scoring process minimizes bias through its transparency and resulting accountability.

The prioritization of research options has important implications for the assignment of available funds that are intended for the control of diarrhoeal disease and the improvement of child health. This approach to improve the decision-making process has steered away from informal meetings of experts that were classically used because of concerns of decision-making flaws resulting from both group dynamics (i.e. groupthink) (11) and vested interest in the development of programmes in the particular field of interest of the expert. The Delphi process was meant to overcome these shortcomings through anonymity of scorers, structured feedback, and progressive feedback to distill or focus-group opinion. However, the Delphi output lacks the transparency of the CHNRI process, and the reasons behind the scorers' decisions are unclear. While the Delphi thereby works to obtain a consensus, the CHNRI process generates a quantitative output that allows for the calculation of uncertainty in the evaluation of research options through analysis of variance of the individual expert's scores. Furthermore, the CHNRI process elucidates the specific contexts within which the priorities are set (preferably with the investors in health research). It offers an approach to systematic listing of large numbers of research investment options and comparison between options from different research domains using the same set of criteria, thus balancing between high-risk and low-risk options. Its systematic nature in scoring research options decreases individual bias while independent scoring by many experts removes the possibility of a few individuals dominating the decisions on priorities.

One of the shortcomings of this scoring exercise done at this level and applied to a local level is related to problems of the context. When scoring is done, as was the case here, to be broadly applied to the majority of population of the developing world, assumptions are made that may not be truly representative of certain areas. A key example of this relates to issues of deliverability, cost, and sustainability. This criterion is the one most likely to vary between areas with different levels of political stability, public infrastructure, and economic resources. For these reasons, at the regional level, it is worth having a group of experts re-score research options for this particular criterion as this may optimize the overall evaluation of different research options in local contexts. An excel file that facilitates this process is available at http://www.icddrb.org/jhpn.

A second shortcoming of this exercise was the limited availability of selected experts for the exercise. Although 19 experts were invited to participate, only 10 individuals contributed their priority scores. In the future, we would recommend linking the scoring activity to a meeting to facilitate higher participation rates among appropriate experts.

Despite limitations of the CHNRI process, it has several advantages over existing priority-setting techniques. First, the transparency of the process in and of itself can be a roadmap to researchers and potential funding agencies in the area of interest. For example, if experts give a low scores for certain research option relating to issues surrounding deliverability and equity, researchers in the area will be stimulated to develop novel delivery strategies to deliver the intervention to those with the greatest need, and granting agencies can direct programme announcements to fund these proposals. Like the Delphi exercise, it controls the influence of group dynamics, although it does so to a greater extent because scorers are not ‘refocused’ or redirected by serial scoring exercises.

It is clear that the domination of health policy and systems research among those obtaining the higher ranks is a consequence of results (effects on the burden of disease) being expected by 2015. A longer timeframe (e.g. 50 years) might allow more long-term strategic events to obtain higher scores. Furthermore, although the framework for scoring is transparent and systematic and it follows that rational answers to narrowly-formatted questions minimize personal biases, there is a possibility that a different group, composed of more policy-makers and programme officers rather than the group that preformed this exercise, may yield somewhat different results. Despite these limitations, we believe that this priority-setting exercise is a useful guide to investors in health research targeting diarrhoeal disease, who hope to observe measurable results prior to the end-date of the millennium development goals.

## ACKNOWLEDGEMENTS

This study was supported by the Child Health and Nutrition Research Initiative (CHNRI) of the Global Forum for Health Research, under support from the World Bank.
